# Managing a Mega Mass Casualty Event by a Civilian Emergency Medical Services Agency: Lessons From the First Day of the 2023 Hamas-Israel War

**DOI:** 10.3389/ijph.2024.1606907

**Published:** 2024-02-29

**Authors:** Eli Jaffe, Oren Wacht, Nadav Davidovitch, Refael Strugo, Oren Blustein, Ido Rosenblat, Eli Bin, Stav Shapira

**Affiliations:** ^1^ Department of Emergency Medicine, Recanati School for Community Health Professions, Faculty of Health Sciences, Ben-Gurion University of the Negev, Beer Sheva, Israel; ^2^ Magen David Adom (MDA), Tel Aviv, Israel; ^3^ School of Public Health, Faculty of Health Sciences, Ben-Gurion University of the Negev, Beer Sheva, Israel

**Keywords:** emergency medical response, pre-hospital care, mass casualty event (MCE), terrorist attack, healthcare resilience

## Abstract

On 7 October 2023, Israel faced an unexpected attack by Hamas, causing over 1,200 deaths and injuring more than 9,000 individuals. This report delves into the rapid medical response spearheaded by Israel’s civilian Emergency Medical Service, Magen David Adom (MDA), during this crisis. Utilizing data from MDA’s electronic database, 4,097 dispatch records from the day were analyzed. Of these, 39.3% were directly related to the attack. EMS teams faced multiple challenges, including handling an overwhelming number of casualties and navigating active combat zones, which impeded safe access to victims, posed significant risks to teams’ safety, and constrained patient evacuation strategies. This incident underscores the importance of reinforcing healthcare resilience, particularly emphasizing the need for centralizing various aspects of response efforts. These include streamlined communication, national coordination of pre-hospital resources, and systemic management of patient evacuations. Moreover, providing substantial support for EMS personnel, who operated in highly challenging conditions, is imperative.

## Background

On 7 October 2023, Hamas launched an unexpected attack against Israel. This has resulted to date in the death of over 1,200 persons, predominantly civilians, including a significant number of children and elderly. Additionally, over 9,000 people were injured, and at least 240 were taken hostage in Gaza [1]. This attack represents the largest number of Jews killed in a single day since the Holocaust [2]. Yet, the casualties were diverse, encompassing not only Israeli Jews but many Arab-Muslim citizens [3], as well as numerous foreign nationals, notably Nepalese and Thai agricultural workers [4]. Controlling for population size, the immediate mortality impact of this assault was 13 times greater than the 9/11 attack on the United States. An equivalent proportion of casualties in the current U.S. population would have approximated 40,000 deaths [5]. This brief report seeks to detail the immediate medical response by Magen David Adom (MDA), Israel’s national Emergency Medical Service (EMS) and a member of the International Red Cross and Red Crescent Societies, during the initial day of this attack, which saw the most substantial civilian human toll in Israel’s history.

MDA oversees 169 primary stations and dispatch centers distributed across eleven regions in Israel. MDA’s fleet comprises more than 1,400 ambulances, outfitted with both basic and advanced life support equipment, 650 motorcycles, and three evacuation helicopters [6]. These are staffed by 2,500 emergency medical technicians and paramedics, which are further supported by over 25,000 first aid responder volunteers, mobilized through a specialized phone app or emergency calls.

The main aim of this report is to detail the sequence and multitude of medical events as captured and documented by MDA’s command and control system during a mega mass casualty event (MMCE), starting from the onset of the attack at 6:30 AM on a Saturday, which coincided with the Jewish day of rest (Sabbath) and the high holiday of “Simchat Torah,” and continuing through to the end of that day. Furthermore, we highlight the challenges faced by the EMS teams and management.

## Evidence

### Methodology

**FIGURE 1:**
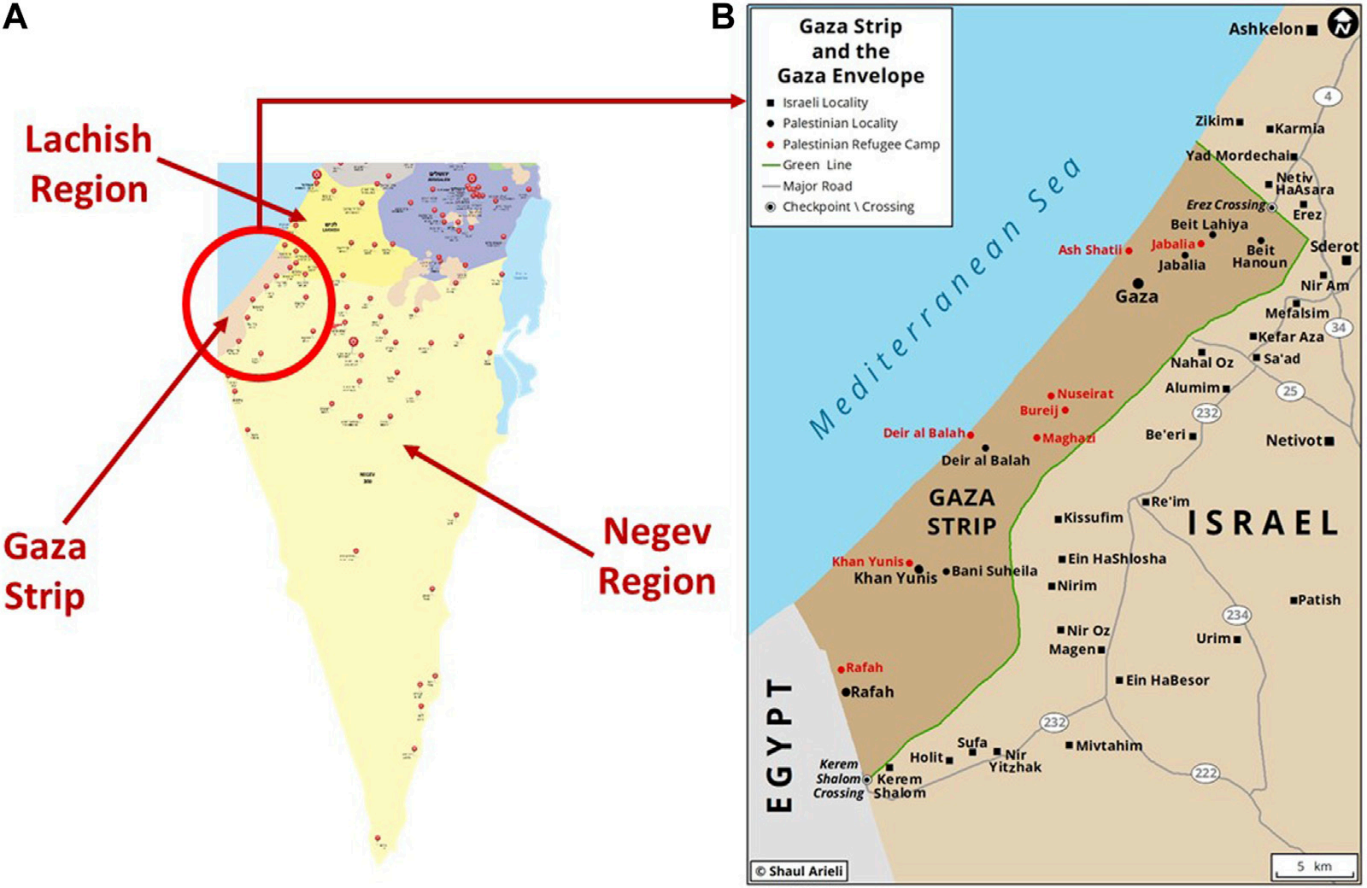
**(A)** is a map of Southern Israel showing the location of Gaza strip and the two MDA regions bordering it: Negev and Lachish. The red markers represent the location of MDA stations (Israel, 2023); **(B)** is an enlarged map of the attack region (Source: Gaza strip and envelope, Shaul Arilei, https://www.shaularieli.com/en/maps/among-other-things/, reproduced with permission).

The current analysis draws from MDA’s electronic database, which encompasses over 95% of the country’s ambulance transports. Dispatchers input the data in real-time. We examined all emergency dispatches from 6:30 a.m. on 7th October when the Hamas-initiated attack began, until the close of the day at 11:59 p.m. This yielded a total of 4,097 dispatch records. For each record, the following data points were extracted: a) dispatch timestamp, b) dispatch code–indicating the nature of the event based on MDA’s established coding system (e.g., injury following rocket hit, terror attack, etc.), c) medical condition—this represents the type and severity of the injury, based on preliminary assessment, and d) incident location, which was determined both by the provided address and its designation within MDA’s eleven geographical dispatch zones. Southern Israel, where the attack transpired, comprises two of these zones: Negev and Lachish. Both zones share a border with the Gaza strip [7]. Figure 1 provides a detailed map highlighting the area affected by the attack and the relevant MDA zones.

We employed descriptive statistics to delineate the volume and characteristics of events where MDA personnel delivered pre-hospital medical assistance. Additionally, we analyzed the event’s dynamics over both time and space. Each record was categorized either as an attack-related event (e.g., dispatches due to rocket attacks or violent actions such as shootings) or as routine, non-terror related dispatches.

### Findings

#### Dispatch Records

Among the 4,097 dispatches conducted during 7th October 1,612 (39.3%) were attributed directly to the attack. Among those—893 (21.8%) represent casualties from rocket impacts, ensuing damage, and fires; 686 (16.7%) are related to violent acts such as shootings. Thirty- three (0.8%) represent specific cases involving casualty transportation to or from helicopter landing sites—primarily for the transfer of trauma patients from the attack to distant hospitals in central or northern Israel. A breakdown of these 1,612 cases by severity reveals 512 incidents involving severe injuries or multiple trauma (32.7%); 170 with minor injuries (10.5%); and 118 characterized by anxiety or stress reactions (7.3%). Only two dispatches fell under the “Incident without casualties” classification.

**FIGURE 2:**
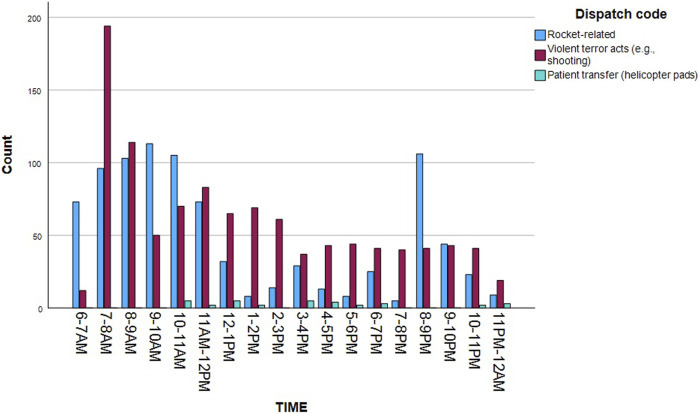
Attack-related dispatches (*n* = 1,612) according to type of event/code (Israel, 2023).

Figure 2 illustrates the distribution of attack-related dispatches throughout the day. A critical point to consider is the discrepancy between the count of dispatches and the actual number of casualties addressed at each incident location. In the context of this MMCE, MDA ambulance teams frequently tended to multiple casualties at a single incident location. This pattern extended to the evacuation and subsequent hospital transfer numbers where individual ambulances often transported 4–7 casualties simultaneously. Consequently, this distinction can explain the difference between the number of ambulance dispatches and the 2,290 attack-related hospital admissions reported by the Israeli Ministry of Health on 7th October. Furthermore, the discrepancy may also stem from other factors, such as patients’ self-evacuation.

Of the total dispatches, 2,485 (60.7%) pertained to routine non-terror activities. This encompasses various medical conditions (*n* = 1,687; 70.3%), vehicular accidents (*n* = 93, 3.9%), and childbirth incidents (*n* = 62, 2.6%). A minimal number of dispatches were linked to the secondary transportation of attack casualties, moving them from southern hospitals to those in central and northern Israel (*n* = 9).

Worth mentioning is that for the 39 Saturdays preceding 7th October 2023, the MDA database recorded an average of 5,598 dispatch calls. In comparison, there was a 26.8% reduction in the number of dispatches on the initial day of the attack. This decrease likely resulted from a drop in routine calls, as the attack led people without urgent medical needs to refrain from contacting EMS—a contrast to typical weekends. Significantly, and as also noted above, the calls received on 7th October typically involved multiple casualties at each location, contrasting with routine calls that usually concern a single individual. Therefore, the reduction in call volume does not imply fewer casualties; in fact, it suggests the opposite due to the nature of the incidents on that day.

## Policy Options and Recommendations

The events of 7th October resulted in an unprecedented influx of fatalities and casualties in Israel. At the date of writing, almost 4 months post-attack, the processes of victim identification, burial, and continued medical care are yet to be complete. This MMCE, largely condensed into a single day, surpassed even the direst predictions for such an incident. Despite Israel’s ingrained culture of emergency preparedness, the healthcare system, especially in the southern region, was tested to its extremes. Both pre-hospital (EMS) and hospitalization facilities were severely strained. Specifically, Soroka Medical Center (the only Level 1 trauma center in the South) received 572 casualties between 7:30 a.m. and 9 p.m., with a total of 676 patients within the first 24 h [8]. Barzilai Medical Center (the second largest hospital in the region) treated over 420 casualties. Overall, Israeli hospitals received a total of 2,290 injured individuals on 7th October alone.

EMS teams faced multifaceted challenges, which extended beyond the sheer volume of dispatch calls and injuries. As the events unfolded on 7th October, The MDA southern dispatch center, coordinated by the central national command and communication system, added dispatchers and mobilized manpower and ambulances from all across Israel to assist in response efforts. At the onset of the attack (6:30 a.m.), roughly 400 manned ambulances across Israel stood ready to respond. Within just 30 min of the event’s commencement, an additional 560 ambulances were staffed and on standby. By the 48-min mark, the count surged to a total of 1,200 manned ambulances poised to address the crisis. These included specialized response vehicles equipped for mass casualty events and which have transport capability of up to four casualties at once. MDA’s operations through a National Command and Control Center was pivotal in orchestrating this rapid and large-scale mobilization of resources. This underscores the advantages of centralized management in coordinating pre-hospital disaster response to large-scale events.

However, access to many casualties was obstructed due to Hamas militants having occupied entire villages and towns. This situation forced EMS teams into extremely precarious positions, having to weigh the urgent need to save lives against the substantial risk to their own safety in active combat zones, which included dense rocket fire and direct gunfire. In planning mass casualty scenarios, MDA had not anticipated one in which EMS would be unable to evacuate injured patients within Israel’s territory due to ongoing hostile activity. Consequently, “pop-up” first aid stations were established at select MDA stations and along major roads outside the targeted villages and towns, waiting until it was safe to move patients to hospitals. In certain situations, the Israeli Defense Forces (IDF) used military vehicles, and civilians even used their own cars, to ferry casualties from attacked zones within villages and towns to EMS teams stationed at key intersections just beyond the zones of conflict. The ensuing chaos frequently resulted in significant delays in administering essential medical care. Such delays caused avoidable fatalities in certain instances, while in others, they led to medical complications. For example, several individuals who had tourniquets applied for extended periods experienced limb ischemia, necessitating subsequent amputation.

Medical interventions were anchored in the “Scoop and run” strategy [9] focusing on immediate life-saving measures such as hemorrhage control and pain management. If a trained professional was on site, advanced life support measures, including tranexemic acid (TXA), freeze dried plasma (FDP), and occasionally blood transfusions, were administered. The choice to adopt a “Scoop and run” approach, as opposed to the “Stay and play” strategy, proved in this case to be the best practice given the sheer number of casualties in the current event and the danger of providing medical care under fire in unsafe environments. This approach allowed EMS teams to execute multiple rounds of casualty collection and expedite their transport to definitive care. The efficacy of this approach was further bolstered by efficient communication and coordination during the handover in the pre-hospital/hospitals interface, ensuring seamless continuity of care.

Patient transport posed another significant challenge and dilemma. As noted above, initial evacuations from the scene primarily targeted the two primary southern hospitals, Soroka and Barzilai Medical Centers, which were quickly inundated with casualties [8, 10, 11]. Secondary transfer, intended to redistribute patients and reduce strain on these centers, was sporadic and primarily employed in the latter part of the day. The decision to evacuate casualties to closer medical centers has two main benefits: it ensured rapid, lifesaving care for the severely injured while also preserving crucial evacuation resources like ambulances. However, this approach risked overloading these hospitals and potentially delaying treatment for less critical injuries. The complexity of patient evacuation and transportation constraints are crucial aspects of medical response efforts in mass casualty events [12, 13]. Future response plans must address these evacuation challenges and weigh the potential risks and benefits concerning the availability of resources and existing capacities of all responding organizations and institutions, as well as patient outcomes, both immediate and long-term [14]. Therefore, it is recommended that a national mechanism under the Ministry of Health jurisdiction be established for the central management of patients’ primary and secondary transfers, adhering to the aforementioned criteria. This topic warrants further in-depth research, integrating data from pre-hospital, hospital, and community post-hospitalization phases. All relevant data are accessible to the Ministry of Health’s evaluation committee, which was recently established to investigate the medical response to the 7th October attack.

In closing, the valor of the EMS teams, operating under daunting conditions and personal risk, highlights the critical need to prioritize their wellbeing by offering support mechanisms, and mitigating potential post-traumatic stress reactions, which are prevalent among first responders in terror incidents [15]. As well, hospital doctors performed with great initiative and under trying conditions [10]. Insights from their experience, along with the lessons gleaned, should inform and refine our emergency preparedness plans. Doing so is pivotal in fortifying the resilience of any health system to navigate both present and forthcoming challenges. Other countries would do well do study Israel’s response to this mass terrorist event as it is unlikely to be the last in this troubled world.

### Conclusions

The 7th October attack highlights the necessity of enhancing healthcare resilience during large-scale emergencies. A key aspect of this resilience is adopting a centralized approach in managing response efforts. This entails establishing streamlined communication channels for efficient information exchange between emergency response entities, national coordination of pre-hospital resources for swift EMS deployment, and systemic management of both primary and secondary patient evacuations to optimize patient care and resource allocation. Supporting EMS personnel’s wellbeing and preparedness are essential for sustaining the high level of emergency response required in such critical situations. Implementing these strategies will fortify the healthcare system against future emergencies.
